# Biofeedback and Exercise Load Affect Accuracy of Tongue Strength Exercise Performance

**DOI:** 10.1007/s00455-024-10751-w

**Published:** 2024-08-18

**Authors:** Erin Kamarunas, Kelsey Murray, Teresa Drulia, Sarah Szynkiewicz, Lindsay Griffin, Rachel Mulheren

**Affiliations:** 1 Department of Communication Sciences and Disorders, James Madison University, 235 MLK Jr. Way, MSC 4304, Harrisonburg, VA 22807 USA; 2 Davies School of Communication Sciences and Disorders, Texas Christian University, Fort Worth, TX USA; 3 Department of Communication Sciences and Disorders, Samford University, Birmingham, AL USA; 4 Department of Communication Sciences and Disorders, Emerson College, Boston, MA USA; 5 Department of Psychological Sciences, Case Western Reserve University, Cleveland, OH USA

**Keywords:** Lingual, Tongue, Tongueometer, IOPI, Exercise dosing, Exercise accuracy, Swallowing, Dysphagia, Biofeedback, Oral manometry

## Abstract

Rehabilitative exercises require precise movement coordination and target accuracy for optimal effectiveness. This paper explores the impact of tongue strength exercises (TSE) performance accuracy on exercise outcomes, adherence, and participant confidence and motivation. An 8-week randomized clinical trial included 84 typically aging participants divided into four groups defined by access to biofeedback (present/absent) and TSE intensity dosing (maximal/submaximal) during a home exercise program (HEP). Retention, training, and HEP accuracy were tracked at biweekly visits and during HEP for participants with access to a biofeedback device. Associations with tongue strength outcomes, participant factors, biofeedback, and intensity dosing were analyzed. Exercise accuracy measures did not contribute to tongue strength outcomes at the end of 8 weeks. Increased training accuracy (less practice required to achieve competency) was associated with higher participant confidence and better adherence to the HEP. The presence of biofeedback was associated with reduced adherence but better retention accuracy, while maximal intensity was associated with improvements in all accuracy measures compared to submaximal intensity exercise. These findings in typically aging participants suggest the need for tailored approaches in swallowing-related exercise programs, given the effects of biofeedback and exercise intensity on motor learning and exercise retention. Accuracy performance and its effect on clinical outcomes warrants study in clinical populations with dysphagia and with various rehabilitative approaches.

Trial Registration Clincialtrials.gov: NCT04809558

## Introduction

Many of the rehabilitative maneuvers that speech-language pathologists (SLPs) use in treatment plans are novel movement sequences and require specific training and verification of accuracy. The accuracy with which exercises are completed during and after initial training may impact their effectiveness, functional improvement, and utilization of resources [1]; in other words, if an exercise is not completed according to the protocol instructions or with sufficient intensity, it may not yield the same outcomes as an accurately executed exercise [2, 3]. This aligns with the experience-dependent plasticity principle of specificity, such that changes in neural plasticity and associated behaviors depend on the nature of training [4]. Accuracy checks of swallowing-related exercises require biofeedback instrumentation which may not be available or in use. Extrinsic feedback via instrumentation augments internal sensation-perception during a maneuver or exercise, which is particularly relevant for individuals with sensory deficits (i.e., hypoesthesia) [5]. Initial evidence suggests that instrumentation-guided biofeedback improves performance accuracy of swallowing maneuvers during training and that accuracy varies by instrumentation modality [6–9]. How accuracy is defined also differs depending on the aim and execution of the exercise itself as well as the instrumentation used for feedback. For certain maneuvers, accuracy may be defined by kinematic or physiological precision, such as using videofluoroscopy to confirm prolonged laryngeal vestibule closure [9]. Techniques that require increased muscle contraction during less complex movement sequences, such as tongue strength exercises (TSE) or effortful swallowing, may define accuracy as achieving a specific intensity load measured by pressure or muscle contraction amplitude [10].

An emerging body of literature supports the use of TSE to improve reduced tongue strength due to sarcopenia and various etiologies of dysphagia [11–15]. TSE typically involves isometric resistance by pushing the tongue superiorly toward the hard palate but can also include protrusion and lateralization against resistance. Strength training recommendations from the limb literature include a moderate load (60–70% of one repetition maximum or 1RM) with low repetitions (e.g., 8–12) per set for novice individuals, advancing to higher loads (80–100% of 1RM) for advanced training [16]. Endurance training of the limbs typically involves lower loads (40–60% of 1RM) with a higher number of repetitions [16, 17]. However, it is still unclear if limb resistance training principles and recommendations apply to the tongue, a muscular hydrostat. The tongue demonstrates at least some resistance to fatigue [18, 19], and while maximal effort is not typically used in limb strengthening protocols [20], maximal effort for TSE has successfully been implemented in previous studies of novice young and typically aging people [21–24]. There is limited evidence that TSE intensity load does not affect isometric tongue strength outcomes in healthy individuals [25, 26], but the effect of intensity load for patient populations has not yet been explored. Additionally, intensity may affect patient engagement in the rehabilitation process, such as accuracy in approximating pressure targets and adherence to the exercise program. Patients tend to be less adherent to higher intensities of gross motor exercise, which results in less exercise being completed [27]. Higher intensities may also be more difficult to achieve during successive repetitions. Van den Steen and colleagues (2019) reported participants had less success in achieving the target pressure as TSE intensity increased. Other studies using variable accuracy targets (e.g., 20–90% of 1RM) have reported mixed gains in strength and function, but did not report how successful participants were at exerting precise amounts of tongue pressure to match the target [14, 28, 29]. Due to a lack of data, it is unclear how intensity load may affect accuracy to exercise target and how accuracy may impact tongue strength outcomes.

Movement accuracy is impacted by knowledge of results and performance [30]. Knowing how closely a target intensity is approximated during TSE requires biofeedback instrumentation, typically through oral manometry. For example, a person completing *maximal* TSE needs to know that they are repeatedly achieving maximal intensity over a set of consecutive repetitions. Submaximal targets may require a longer acquisition period (practice) with knowledge of results to refine the motor plan before retention of the correct movement intensity can occur. The modality of biofeedback may impact knowledge of performance and thus accuracy. Ultrasound biofeedback doubled post-training accuracy of the Mendelsohn maneuver and improved retention after one week compared to using surface electromyography (sEMG) [7]. Participants post-stroke who trained on a volitional laryngeal closure maneuver were significantly more accurate when using videofluoroscopy (VF) or a combination of VF and sEMG compared to sEMG alone [9]. There is clear evidence to support using biofeedback, when available, to train and complete rehabilitative swallowing maneuvers. However, it is unclear whether TSE require biofeedback for accuracy and retention and to what extent exercise intensity modulates this effect. Furthermore, internal factors such as self-efficacy may affect exercise accuracy [31]. Consideration of performance accuracy and the factors that affect it may be especially important in outpatient settings where home exercise programs are utilized and access to clinician feedback is intermittent.

The aims of this paper are to 1) determine which participant factors, including exercise accuracy measures, sex, and age, are predictive of oral pressure gains, confidence, motivation, and adherence to the exercise program and 2) determine if exercise conditions including biofeedback and exercise intensity have an effect on exercise accuracy measures. It was hypothesized that 1a) exercise accuracy would be predictive of oral pressure changes with increased accuracy being associated with increased gains, 1b) increased accuracy and the presence of biofeedback would also be predictive of increased participant motivation, confidence, and adherence; exercise intensity was predicted to not affect motivation, confidence, or adherence; and 2) the presence of biofeedback and maximal exercise intensity would improve exercise accuracy measures.

## Methods

This data is from a larger, multisite study and the main findings have been previously published [26]. This paper represents a distinct dataset that while related to the original publication, is unique in content and focus. This study was registered as a clinical trial (NCT04809558).

### Participants

A university institutional review board approved this study. All participants voluntarily signed a written informed consent before starting the study. Community-dwelling participants were typically aging adults aged 55 years and older who did not have swallowing difficulty. Inclusion criteria included a score of < 3 on the Eating Assessment Tool (EAT-10) [32], a score of > 24 on the Mini-Mental State Examination (MMSE) [33], and intact oral structures and lingual range of motion as determined by an informal oral mechanism screen. Exclusion criteria included a history of seizures, mandibular pain, oral surgery that affected the structure of the mouth or tongue (not including routine dental surgery), neurogenic diseases, or any other health condition that may affect tongue strength or the ability to swallow. Medical history was determined by participant self-report during the screening process.

A power analysis based on tongue strength outcomes as the primary study aim [26] was completed at the beginning of the study and determined that a sample of 100 would provide a power of 0.99. Power analyses were repeated throughout data collection and participant recruitment stopped when the target power was achieved for those outcome measures. Table 1 details participant enrollment and demographics.

**Table 1:** Group descriptions and demographics

		Group				
		Max/-BF	SubM/-BF	Max/ + BF	SubM/ + BF	
Brief description		Maximal intensity; no biofeedback	Submaximal, progressive intensity; no biofeedback	Maximal intensity; biofeedback used	Submaximal, progressive intensity; biofeedback used	
Training details	Intensity	100% 1RM*	Weeks 1–2: 50% 1RM Weeks 3–4: 60% 1RM* Weeks 5–6: 70% 1RM* Weeks 7–8: 80% 1RM*	100% 1RM*	Weeks 1–2: 50% 1RM Weeks 3–4: 60% 1RM* Weeks 5–6: 70% 1RM* Weeks 7–8: 80% 1RM*	
	Biofeed-back	Biofeedback used to train to the exercise, but not used during HEP	Biofeedback used to train to the exercise, but not used during HEP	Biofeedback used to train to the exercise AND to guide exercises during HEP	Biofeedback used to train to the exercise AND to guide exercises during HEP	
Demographics						Sig
Age, x̄(SD)		64.3 (6.9)	66.4 (7.9)	63.4 (5.6)	61.6 (5.2)	*p* = 0.12
Sex		7 M, 14 F	5 M, 17 F	4 M, 17 F	8 M, 12 F	*p* = 0.42
Screening results						
EAT-10, x̄(SD)		0.33 (.48)	0.5 (.8)	0.24 (.5)	0.25 (.6)	*p* = 0.47
MMSE, x̄(SD)		29.4 (.6)	29.2 (1.2)	29.6 (.8)	29.2 (1.2)	*p* = 0.56

*SubM* submaximal, *Max* maximal, *HEP* home exercise program, *1RM* one repetition maximum, *BF* biofeedback, *M* male, *F* female

*Recalculated every 2 weeks

### Design

The design of the study was a randomized clinical trial. Visits were completed in each site investigator’s private university laboratory. Participants were randomly assigned to four different TSE treatment groups. Exercise groups differed by exercise intensity (100% of 1RM or 50–80% of 1RM) and biofeedback guidance (present/absent) (Table 1). Details about the exercises and training schedule can be found in previous work [26]. Briefly, TSE instruction began during a baseline visit and was repeated at check-ins every two weeks for eight weeks. Tongue pressures were measured at every visit during maximal isometric pushing, regular saliva swallows, and effortful saliva swallows [26].

### Training

Participants were trained to their TSE targets during the initial visit and retrained to new targets during the check-in visits every two weeks. The training was completed using a Tongueometer™ device with the Tongueometer™ application on a Lenovo tablet. A small piece of tape was added to the tongue bulb tubing to give participants sensory feedback as to the position of the bulb in their mouths and to replicate the approximate same placement across visits. Participants were instructed to push their tongue up against the tongue bulb until their target was reached, hold it for 2 s, then relax their tongue. They practiced this several times until they understood the exercise technique.

Competency to the exercise was established for the two groups who used biofeedback during home exercise practice (HEP) by completing eight consecutive tongue exercise trials in which the target pressure was hit within ± 25% of the goal while viewing the biofeedback. Following unsuccessful trials, verbal feedback was given, and the participant continued to practice until eight consecutive successful trials were completed. Separately they were trained on how to use the Tongueometer™ application and the tablet and were given written instructions for both.

For the two groups who did not use biofeedback during HEP, competency was established by completing eight consecutive trials, which consisted of three consecutive trials within ± 25% of the goal *with* visual biofeedback and then five consecutive trials at ± 25% of the goal *without* visual biofeedback. If any of the eight trials were not within 25% of the target, verbal feedback was given (e.g., “Try pushing harder/softer”) and the process was repeated (three trials with biofeedback, five trials without biofeedback) until eight consecutive successful trials were completed. Participants were cued to pay attention to how hard they had to push to reach the target, so this could be repeated during HEP without biofeedback. Competency was established every two weeks for all groups when the target pressure was recalculated.

### Home Exercise Program

All participants were requested to complete their assigned TSE across 3 sets of 10 repetitions, 3 days/week for 8 weeks, with the instruction to complete all of the daily sets at one time and for the three days to be nonconsecutive, if possible. A written log was provided to participants to document all home exercises. The completed written log was used to calculate adherence to their program, defined as the percentage of repetitions completed relative to the number prescribed. Participants who completed their HEP without biofeedback were given a sealed air-filled tongue bulb to use during exercise in the same manner as those using biofeedback, but it was not connected to a device and they documented each repetition completed. Participants with biofeedback were requested to also document if they were within the target range (± 25%) or not for each repetition completed at home.

Outcome Measures.

### Accuracy

Accuracy to target tongue pressures was measured in three distinct ways across various time points: 1) *retention accuracy* at the beginning of check-ins 2) *training accuracy* during competency trials as part of training visits and 3) *HEP accuracy* during home exercises (only for the groups using biofeedback). The definitions of the accuracy outcomes are provided below and in Table 2.

**Table 2:** Accuracy measure definitions

Measure	Description	Definition
Retention accuracy	Taken at the beginning of each check-in. following two weeks of HEP, participants were asked to demonstrate the TSE at the target intensity load, which reflected how well they remembered the instruction from two weeks prior. A smaller difference between the two values indicated better retention accuracy	$$|\text{ T}arget pressure value -Actual pressure value |$$
Training accuracy	Taken during the training portion of each session including the initial session and all check ins. Participants practiced the TSE with the goal of hitting the specific pressure target on 8 consecutive practices. A smaller number indicated better training accuracy	# of trials required to achieve 8 consecutive trials within the target pressure range. The smallest number possible was 8
HEP accuracy	During the home practice, participants who had biofeedback documented whether or not they hit the preset pressure target in a binary fashion (yes/no). A larger percentage indicated better HEP accuracy	$$\frac{\# of trials within the target range}{\# of trials attempted }\times 100$$
Adherence	The percentage of completed HEP based on the dosage requested (3 sets of 10 repetitions 3x/week). A larger percentage indicated better adherence	$$\frac{\# of completed trials}{Total dosage }\times 100$$

*HEP* home exercise practice, *TSE* tongue strength exercise

At the beginning of check-ins (every two weeks once the exercise protocol was started), the participants were asked to demonstrate the assigned tongue exercise using the Tongueometer™ device across three trials in the same manner they completed it at home and tongue pressure values were recorded. Retention accuracy was measured by taking the absolute value of the mean difference between the actual tongue pressure values and the target pressure value. Therefore, larger values indicate worse retention accuracy.

Training accuracy was measured during competency trials by documenting the tongue pressure value of competency trials and determining the percentage that fell within the target tongue pressure range. The number of pushes required to achieve competency as defined by the study protocol were also documented and reported in aggregate.

Measurement of accuracy during the HEP was feasible only for the biofeedback groups with immediate access to the pressure values from their Tongueometer™. Participants using biofeedback documented on their practice log whether each exercise repetition was within the target range in a binary fashion for simplicity. Data recorded by the Tongueometer was not used in the analysis due to discrepancies in how completed trials were defined. For example, the participants were instructed that if they missed the preset target range to mark the trial as completed but missed. The Tongueometer™ tracked data counts the trials as completed only if they meet the target so target undershoots are not documented by the software and therefore are missing data. On the exercise log, if the target was missed, the direction of the inaccuracy (undershot/overshot) was not documented. HEP accuracy was calculated in proportion to the number of completed trials and reported in percentage. As all accuracy measures were collected multiple times across the treatment period, these values were aggregated for each accuracy type for every participant.

### Participant Motivation and Confidence

Following training at the baseline visit and re-training during each check-in, all participants were asked to respond to the statements, “I am confident that this type of exercise will improve my tongue strength,” and, “I am motivated to complete these exercises”. Each rating was completed using a 100-mm visual analog scale (VAS). The confidence scale was anchored on the left with “not confident at all” and the on the right with “extremely confident”. The motivation scale was anchored on the left with “not motivated at all” and the right with “extremely motivated”. The investigator who administered these scales was often the one who provided the exercise training. This reflects logistic necessity. Ratings were then measured and documented as a 0–100 score. Confidence and motivation measures were aggregated across time for each participant.

### Other Outcomes

Adherence to the tongue strength home exercise program was tracked via participant log that was turned in every two weeks. Adherence was calculated as the mean ratio of completed exercise to total requested exercise (%). Changes in tongue strength were tracked using the Iowa Oral Pressure Instrument (IOPI) at baseline and each check in. While the Tongueometer was used during training and the HEP, an IOPI was used separately for outcome data as described in Szynkiewicz et al. (2023). There were several reasons for this including that there is established normative data for the IOPI and that while the two devices likely both measure tongue pressure in a similar way, they may not have comparable pressure values [34–36]. Participants completed five trials each of maximum isometric pressures, regular saliva swallows, and effortful saliva swallows, all completed with anterior bulb placement as previously detailed [26]. A small piece of tape was also used to ensure standardization of placement within and between sessions using the same process as was completed with the Tongueometer™ bulb. Oral pressure change for the current analysis was calculated as the mean change in oral pressure from baseline to the last visit across all three tasks. These were collapsed to reduce the number of comparisons made. The group comparisons for adherence and changes in tongue strength have been previously reported [26] and are not repeated here. Both adherence and oral pressure changes were used in the statistical analyses to determine associations with the accuracy measures, motivation, and confidence. Descriptives and group comparisons of this data will not be presented here, and the reader is referred to previous publications for this information [26].

### Statistical Analyses

All data analyses were completed using IBM SPSS Statistics (Version 28.0). Accuracy measures descriptives are reported. The effect of biofeedback and exercise intensity level on exercise accuracy measures was examined with Mann–Whitney U tests given the non-normal distribution.

Backward linear regression analysis was used to identify the most significant predictors of tongue strength gains, confidence, motivation, and adherence. This method allowed for systematic removal of non-significant variables from the regression model until only the most influential predictors remained. The first model tested if accuracy measures, adherence, confidence, motivation, age, or sex significantly predicted gains in tongue strength post-treatment. The predictive value of biofeedback and exercise intensity was not included in this model since this has previously been reported [26].

Additional backward regression models were completed to test if accuracy measures, sex, age, biofeedback, or exercise intensity predicted participant confidence, motivation, and adherence to the exercise program. The backward elimination method had a criterion set at a probability of F-to-remove > / = 0.10 and an alpha level of 0.05. None of the accuracy outcome data were normally distributed, as assessed by the Shapiro–Wilk’s test (*p* < 0.05) and heteroscedasticity was observed for most dependent variables. Given the uncertainty of interpreting the models’ significance due to the data’s non-normal distribution, bootstrapping was used (1000 samples), which allowed for resilience to breaches of assumptions like normality and homoscedasticity. Furthermore, it enabled the calculation of bias-corrected and accelerated confidence intervals and hypothesis test statistics for regression coefficients, all without the need for asymptotic assumptions. The alpha was set to 0.05 following Bonferroni correction for multiple comparisons.

## Results

### Participant Demographics

**Fig. 1:**
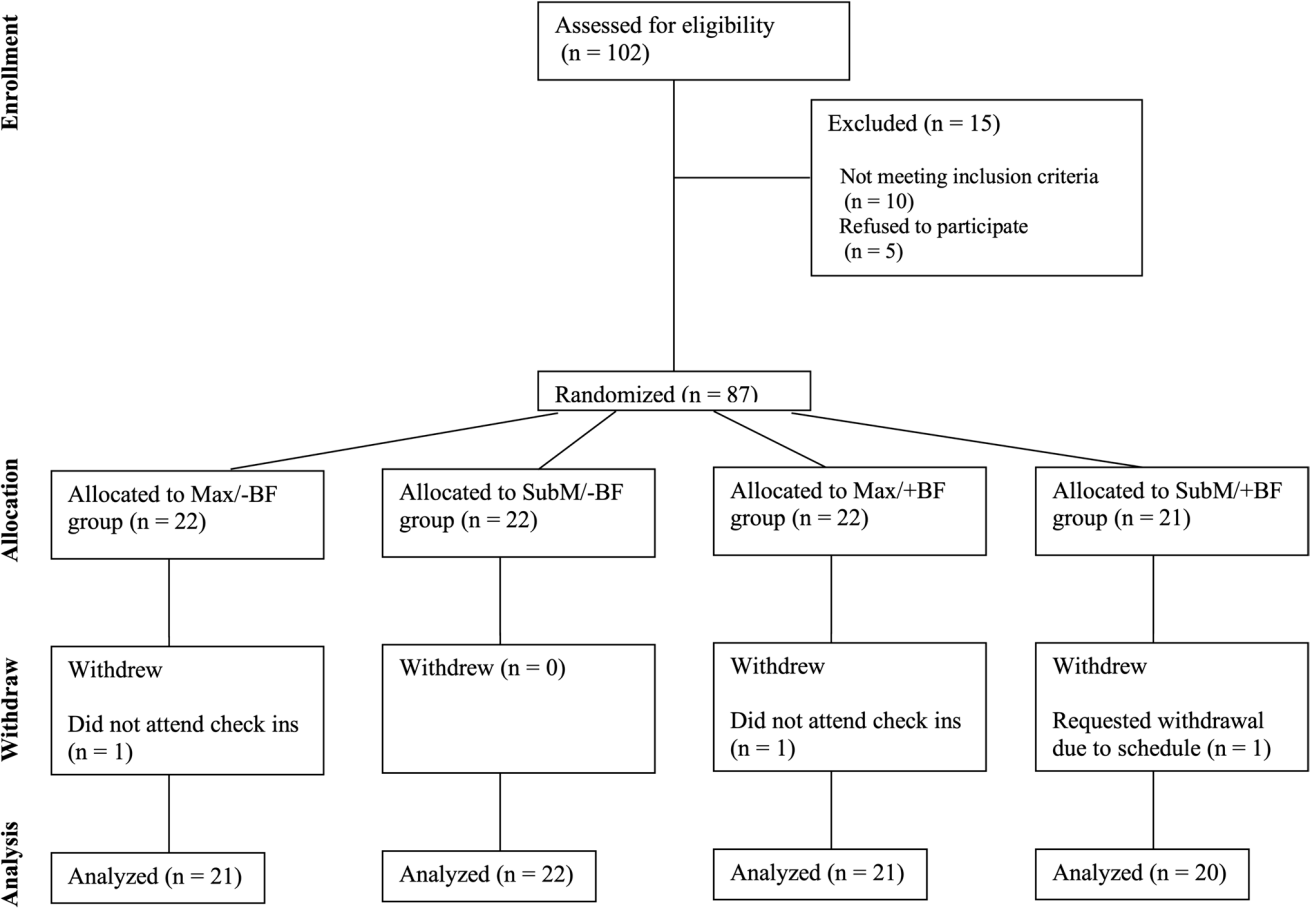
CONSORT diagram showing the flow of participants through each stage of a randomized trial

Recruitment was open from June 2021 to August 2022. Eighty-seven participants were enrolled in the study. Three participants did not complete the protocol and were not included in the final data analyses. Reasons for withdrawal included two participants not returning for scheduled study visits and one participant having schedule conflicts for continued participation. A CONSORT flow diagram is provided in Fig. 1. The final dataset included eighty-four participants (60 female), with a mean age of 64 years (range: 55–82 years). Other participant demographics are presented by group in Table 1. Oral pressure changes pre- and post-treatment have been reported previously [26] and will not be repeated here.

### Accuracy Measures and Effect of Biofeedback and Exercise Intensity

Retention (from instruction two weeks prior) accuracy data was collected from 84 participants, with complete datasets across four visits for 75 participants. Mean retention accuracy was 5.8 ± 4.8 kPa across groups (range: 0.01–28.5 kPa), meaning that upon return two weeks after training, participants were, on average, approximately 6 kPa above or below their target pressure. Mean retention accuracy was statistically significantly improved for participants using biofeedback (5.1 ± 2.9) than those without (6.7 ± 2.8), *U* = 527.0, *z* = −3.2, adj *p* = 0.01, effect size *r* = 0.35 (lower scores indicate closer to target pressure). Mean retention accuracy was also improved for participants with maximum intensity exercise (5.1 ± 2.7) compared to submaximal exercise (6.7 ± 3.0), *U* = 1190.0, *z* = 2.8, adj *p* = 0.03, effect size *r* = 0.31. Using Cohen’s definitions, these reflect medium effect sizes [37].

Training (during session) accuracy was collected from 84 participants, with complete datasets across four visits for 77 participants. Mean training accuracy across groups was 96.4% ± 7.6%, meaning that on average, 96% of participants’ practice pushes were within the target range. Mean training length was 10.2 ± 8.4 pushes, indicating that it required 10 pushes to establish competency to the exercise (defined as eight consecutive pushes within the acceptable range). Training pushes were, on average, 4.0 ± 1.9 kPa above or below the target pressure. Mean training accuracy did not differ between participants using biofeedback (10.3 ± 8.4) than those without (9.9 ± 2.8), *U* = 654.0, *z* = −2.1, adj *p* = 0.17, effect size *r* = 0.23. This indicates a small to medium effect size. However, training accuracy was improved for participants with maximum intensity exercise (8.9 ± 2.0) compared to submaximal exercise (11.4 ± 8.3), *U* = 1333.0, *z* = 4.22, adj *p* = 0.005, effect size *r* = 0.46 (lower scores indicate less training required to achieve exercise competency). This reflects a medium to large effect size.

Self-reported HEP accuracy (meeting the pressure target) from 40 participants was collected from participants in the biofeedback groups who received immediate feedback on their TSE performance at home. Complete datasets across all eight weeks of HEP were available for 35 participants. The mean HEP accuracy was 95.5% ± 8.1%, meaning that 95% of HEP pushes fell within the target range. HEP accuracy was statistically significantly improved for participants completing maximum intensity exercise 97.4 ± 4.7) compared to those completing submaximal exercise (93.8 ± 5.1), *U* = 83.0, *z* = −3.2, adj *p* = 0.005, effect size *r* = 0.51. This indicates a large effect size.

### Predictors of Oral Pressure Change

A complete dataset for oral pressure changes from baseline to the end of the study for 84 participants was included. A backward linear regression was run to predict oral pressure outcomes from participant confidence, motivation, adherence to the program, training accuracy, HEP accuracy, and retention. Sex and age were included in the model. There was linearity and heteroscedasticity, as assessed by studentized residuals plotted against the predicted values. There was no evidence of multicollinearity, as assessed by tolerance values greater than 0.1. The assumption of normality was not met. The final backward regression model revealed that none of the models that included accuracy measures were better at predicting oral pressure outcomes than the model that included age alone. Of the included variables, age was the only predictor of oral pressure change for healthy participants (β = -0.41, *SE* = 0.11, *p* = 0.002). There was a weak negative correlation between age and oral pressure change (r_s_ = −0.35). Accuracy measures, adherence, confidence, motivation, and sex did not significantly impact tongue strength gains in our model. The final regression model accounted for 8% of the variance in oral pressure change, as indicated by the adjusted R-squared value. The model’s F-statistic was statistically significant (*F*(1, 83) = 7.9, *p* < 0.01), suggesting that the predictor, age, explained a significant proportion of the variance in oral pressure change. The 95% bootstrap confidence interval for the β of age was (-0.64, -0.22). The fitted regression model was Y = 34.97 + [−0.41] * age.

### Predictors of Participant Confidence

Confidence measures were gathered for 84 participants with complete data sets across all visits for 73 participants. Mean confidence across visits was 84 ± 15.8 (out of 100).

A backward linear regression was run to predict participant confidence from adherence to the program, training accuracy, HEP accuracy, retention, biofeedback, exercise intensity, sex, and age. There was linearity and heteroscedasticity, as assessed by studentized residuals plotted against the predicted values. There was no evidence of multicollinearity, as assessed by tolerance values greater than 0.1. The assumption of normality was not met. The final backward regression model revealed that training accuracy (β = −0.46, *SE* = 0.38, *p* < 0.05) was the strongest and only predictor of participant confidence. Retention accuracy, HEP accuracy, biofeedback, exercise intensity, adherence, age, and sex were not found to have a significant impact on participant confidence in our model. The final regression model accounted for 4.5% of the variance in confidence, as indicated by the adjusted R-squared value. The model’s F-statistic was statistically significant (*F*(1, 83) = 4.9, *p* < 0.05), suggesting that the predictor, training accuracy, explained a significant proportion of the variance in participant confidence. The 95% bootstrap confidence interval for the β of training accuracy was (-1.4, -0.35). The fitted regression model was Y = 89.3 + [−0.46] * training accuracy.

### Predictors of Participant Motivation

Motivation measures were available from 84 participants with complete datasets across all visits for 74 participants. Mean motivation was 90 ± 12 (out of 100).

A backward linear regression was run to predict participant motivation from adherence to the program, training accuracy, HEP accuracy, biofeedback, exercise intensity, sex, and age. There was linearity and heteroscedasticity, as assessed by studentized residuals plotted against the predicted values. There was no evidence of multicollinearity, as assessed by tolerance values greater than 0.1. The assumption of normality was not met. There were no significant predictors of participant motivation within the tested variables. The final model included adherence (β = 0.15, *SE* = 0.09, BCa 95% CI (−0.033, 0.29), *p* > 0.05), which did not significantly explain the variance in participant motivation (*F*(1, 80) = 1.98, *p* > 0.05).

### Predictors of Adherence to the Exercise Program

Complete or partial adherence (completion of requested repetitions) data was collected across the 84 participants. Mean adherence was 97.0% ± 10.6%.

A backward linear regression was run to predict adherence to the exercise program from training accuracy, HEP accuracy, retention, biofeedback, exercise intensity, sex, and age. There was linearity and homoscedasticity, as assessed by studentized residuals plotted against the predicted values. There was no evidence of multicollinearity, as assessed by tolerance values greater than 0.1. The assumption of normality was not met. The backward regression model indicated that predictors of adherence included biofeedback (β = −5.2, *SE* = 2.2, *p* < 0.05), age (β = 0.29, *SE* = 0.15, *p* = 0.05), and training accuracy (β = 0.43, *SE* = 0.23, *p* < 0.01). Retention accuracy, HEP accuracy, exercise intensity, and sex were not found to have a significant impact on adherence in the model. However, examination of the bootstrap bias-corrected and accelerated 95% confidence intervals for training accuracy (-0.16, 0.74) and age (-0.08, 0.64) indicated that they were not significant predictors within the model but were independent predictors of adherence (Table 3). Examination of adherence means by biofeedback indicated that participants who completed their program without biofeedback had a mean adherence of 100.0% (BCa 95% CI (97.1, 103.1)) and participants with biofeedback had a mean adherence of 93.9% (BCa 95% CI (90.4, 97.1)).

**Table 3:** Simple regression results for adherence to the exercise program for predictors in the final model

Predictor	*B*	95% CI for *B*^†^		*SE B*	β	*Adj. R*^*2*^	Comparison to null
		*LL*	*UL*				
Biofeedback	−6.1	−10.6	−1.8	2.3	−.28	0.07	*F*(1,80) = 6.9, *p* = .01
Age	0.38	0.08	0.76	0.16	0.24	0.044	*F*(1,80) = 4.7, *p* < .05
Training accuracy	0.41	0.03	1.04	0.22	0.24	0.05	*F*(1,80) = 4.8, *p* < .05

*B* unstandardized regression coefficient, *CI* confidence interval, *LL* lower limit, *UL* upper limit, *SE B* standard error of the coefficient, *β* standardized coefficient, Adj. *R*^*2*^ Adjusted R^2^

^†^bootstrap bias-corrected

## Discussion

For this study of typically aging adults, the effect of tongue strength exercise (TSE) accuracy and other participant factors (e.g., age, sex, confidence, motivation) on tongue strength gains was examined, as well as the effect of biofeedback and exercise intensity on participant performance of TSE (i.e., accuracy). Age was the significant predictor of oral pressure outcomes in this non-dysphagic cohort, which is perhaps unsurprising given that tongue strength is known to decline with age [38, 39], potentially giving older participants an increased likelihood for improvement before a ceiling effect. Age was also a predictor of adherence with adherence increasing with age. Interestingly, adherence rates did not predict improved oral pressure outcomes. While this finding mirrors previous findings of a lack of association between TSE adherence and outcome measures [40], there is literature to support the importance of adherence and dosing in dysphagia rehabilitation [41, 42] and exercise science [43]. None of the accuracy measures taken in this study were associated with improvements in tongue strength. More specifically, training accuracy, retention accuracy, and home exercise program (HEP) accuracy were not predictive of increased tongue pressure generation for maximal isometric pushes, saliva swallows, or effortful saliva swallows following a TSE protocol. This may suggest that, for TSE, exact precision to the target pressure during treatment is not required even if target pressures are consistently undershot.

Motor learning, or relearning as occurs during rehabilitation, requires training to the task. Accuracy of motor performance during both training and execution of the exercise may have ramifications for the effectiveness of the rehabilitation program. Additionally, examination of factors that may affect exercise accuracy, such as biofeedback, exercise intensity, and patient factors (e.g., age, motivation, cognition), may be important considerations for both clinicians and researchers. That accuracy to TSE targets did not affect gains in oral pressure generation may be related to the fact that accuracy rates were relatively high in these typically aging adults, even at the beginning of training. While yet unexplored, it is possible that accuracy to the exercise target may play a larger role in patients with significantly reduced lingual strength and fatigue.

Training accuracy (i.e., how many practice trials the participant required to meet competency) predicted participant confidence and adherence to the program, while exercise intensity did not affect adherence. It is worth considering that the difficulty or ease of swallowing exercise training can have long-lasting effects on how people approach an exercise or rehabilitation program. For example, if rehabilitative exercises seem difficult and take a long time to train, people may have less confidence that they can do the program independently and may be less likely to then follow through on their home program [27]. HEP accuracy and retention did not contribute to confidence, motivation, or adherence. While participants were aware of their HEP accuracy as it was self-documented, retention accuracy measurements were not overtly communicated to participants during check-ins. Therefore, retention performance may not have affected their attitudes and responses to the program in either direction. Of course, more research is needed to determine if these relationships are present in patient populations.

### Biofeedback Effects on Accuracy and Adherence

Biofeedback did not improve training accuracy compared to no biofeedback during training; however, the presence of biofeedback during the TSE training and at home (HEP) improved participants’ ability to recall and achieve tongue pressure targets at in-person visits (retention accuracy). Even if participants are adequately trained to the exercise targets, the internal motor schema for the exercise may fade if continuous extrinsic feedback is not provided during HEP.

While there is extensive research on swallowing rehabilitation methods that include biofeedback [44], there is limited evidence that swallowing treatment programs with biofeedback improve functional outcomes over an equivalent program without biofeedback [5]. The benefit of biofeedback on treatment outcomes may be dependent on the specific exercise and movement goal. For example, if the goal is eliciting greater strength, contraction, and/or pressure, then biofeedback is likely beneficial because it provides a patient with a visual target to reach the goal. Both healthy controls and patients post-stroke produce greater muscle contraction when executing an effortful swallow with sEMG biofeedback compared to without it [10]. While biofeedback did not improve tongue pressure outcomes in an eight-week TSE program, there was a trend toward significance [26]. Two recent studies examining biofeedback in post-stroke patients yielded opposing results. In one study, patients with subacute stroke who completed a five-week swallowing rehabilitation program (effortful swallow, supraglottic swallow, Masako maneuver) with sEMG biofeedback had better pharyngeal clearance and a lower risk of aspiration compared to patients who completed the same program without biofeedback [45]. In contrast, patients with acute stroke who completed a two-week swallow treatment program with sEMG biofeedback showed no advantage in functional outcome measures (e.g. quality of life, feeding route) compared with those without biofeedback [46]. It is possible that the variations in treatment duration and outcome measures explain these differences.

Interestingly, biofeedback had a negative impact on adherence. While this study did not qualitatively explore the reasons for this, it is possible that participants who had biofeedback that required setting up a device prior to their HEP (e.g., charging the battery, syncing with the tablet, troubleshooting) found this process somewhat cumbersome which may have affected their adherence. On the other hand, participants without biofeedback had almost no setup and pushed on a bulb that was not connected to a device, presumably a faster scenario that may have contributed to the improved adherence.

### Exercise Intensity Effects on Accuracy

Participants assigned to maximal TSE had improved accuracy measures over those assigned to submaximal exercises. Training to the pressure target required fewer practice trials for participants with maximal exercises. This is likely related to maximal intensity targets requiring a less complicated internal motor schema compared to submaximal intensity targets, which may also contribute to the improved retention accuracy for these participants. While exercise intensity did not directly predict confidence, these may be indirectly linked since faster time to training accuracy was associated with higher participant confidence.

Maximum exercise intensity also improved home exercise performance accuracy. The maximal intensity group had higher accuracy across the 30 pushes per set compared to the submaximal group, which suggests that the dose of 30 maximal repetitions was not enough exercise to induce lingual fatigue in this non-dysphagic group. There were also no differences in reported fatigue [26] or motivation between maximal and submaximal groups. However, a previous study on TSE intensity load found a reduced ability to achieve exercise targets with maximal loads during treatment sessions. Specifically, participants assigned to maximal intensity loads (100% of 1RM) were approximately 43% successful during TSE practice, while those assigned to submaximal intensities of 80% and 60% of 1RM were 71% and 91% successful at hitting the intensity target, respectively [25]. This disparity is likely related to the repetition dosing, which was 120 pushes per session compared to 30 in this study, which likely contributed to fatigue and reduced accuracy for those attempting maximal intensity [25]. Additionally, there are differing definitions of accuracy—any trial that met at least the minimal target was counted as accurate (e.g., if target was 60%, any pressures ≥ 60% were counted as correct), whereas the current study defined accuracy within a ± 25% range. While achieving a submaximal pressure target may be challenging, maintaining maximal pressure for 100 or more repetitions may not be feasible due to muscle fatigue. Fatigue of the lingual musculature may already be present in patients who present with dysphagia [47], complicating the expectation of repeated maximal effort trials. Additionally, swallow-related fatigue is a significant predictor of dysphagia and malnutrition in older adults [48]. Thus, submaximal targets may be a more feasible starting point for TSE.

### Limitations

There are several limitations to consider which constrain the interpretation of the findings. Although the models reported above were statistically significant, the level of variance accounted for by the various regression models was relatively low (4.4–8.0%). Also, participants were typically aging adults without dysphagia, a formal diagnosis of reduced tongue strength, or cognitive decline. It is unclear how the results of this study might translate to older individuals with dysphagia and reduced tongue strength, and this merits future investigation. Certainly, many of the patients who need dysphagia rehabilitation may also have concomitant cognitive deficits that could limit their ability to complete a rehabilitation program. However, it would be of interest to explore how cognitive decline influences dysphagia rehabilitation selection and training, and how these in turn impact rehabilitation outcomes.

Limitations also exist in the specific group assignments to biofeedback and exercise load intensity. Different modalities of biofeedback (e.g., visual, auditory, display design, device) might yield different outcomes, and this aspect was not addressed. Likewise, the categorization of TSE intensity into two levels may oversimplify the spectrum of exercise intensity. A more nuanced approach to measuring intensity could provide deeper insights. The treatment program only included TSE and therefore the findings can only be applied to this one dysphagia exercise type. While other studies have explored the accuracy of swallowing exercises [6, 8, 9], few have examined the long-term effects of biofeedback [45, 46] or load intensity [25] on swallowing outcomes. No studies have examined how exercise accuracy affects long-term outcomes in a dysphagia rehabilitation program.

The phrasing of the confidence and motivation questions was in a positive direction and may have introduced some bias. Furthermore, the administration of these questions by the investigator who completed the TES training may also have influenced the participant responses and biased the outcome in a more positive direction. Additionally, certain measures, such as HEP accuracy and adherence, were self-reported. This method could introduce bias or inaccuracy in the data, as participants’ reporting might not accurately reflect their actual performance. While the study focused on biofeedback and exercise intensity, other factors that might influence TSE outcomes, such as cognitive status, nutritional status, or overall physical activity level, were not examined. While cognitive status was controlled for, additional individual factors were not. Assessing the impact of these factors in future research could lead to more individualized and effective therapeutic interventions.

## Conclusions

In conclusion, our research underscores the initial stages of considering how dysphagia outcomes are affected by exercise accuracy. While this study explored only one exercise type in non-dysphagic adults, these preliminary results indicate that training ease/difficulty may affect both confidence and adherence. The importance of considering both biofeedback and exercise intensity in TSE programs was also examined. Biofeedback appeared to have a negative impact on adherence but improved exercise retention. Participants completing maximal intensity exercise had improved training, retention, and HEP accuracy, likely related to simpler motor schema compared to submaximal exercises. It should be considered that home exercise program accuracy to TSE may affect tongue pressure gains differently in persons with reduced tongue strength and dysphagia and this should be explored in patient populations. These insights are crucial for clinicians and researchers in developing effective TSE programs. Future research should examine these dynamics to optimize interventions for older individuals and patients with swallowing difficulties.

## Data Availability

The participants of this study did not give written consent for their data to be shared publicly and supporting data is not available.
